# A Novel Approach to Waste Recycling and Dye Removal: Lithium-Functionalized Nanoparticle Zeolites

**DOI:** 10.3390/molecules29194643

**Published:** 2024-09-29

**Authors:** Diana Guaya, Alexis Debut, Jhuliana Campoverde

**Affiliations:** 1 Departmento de Química, Universidad Técnica Particular de Loja, Loja 110107, Ecuador; jncampoverde2@utpl.edu.ec; 2 Centro de Nanociencia Nanotechnología, Universidad de las Fuerzas Armadas ESPE, Sangolquí 171103, Ecuador; apdebut@espe.edu.ec

**Keywords:** methylene blue, lithium, zeolite phases, mining tailings, adsorption, photocatalysis

## Abstract

A zeolitic sample, named MT-ZLSH, was synthesized using mining tailings (MT) as the precursor material, resulting in a structure comprising: Linde type A (LTA) and sodalite-hydroxysodalite (ZLSH). This naming convention reflects the material’s origin and its structural characteristics. The material was further modified by incorporating lithium, producing MT-ZLSH-Li^+^. Physicochemical characterizations were performed, and the material was evaluated for its potential to remove methylene blue (MB) from synthetic wastewater through adsorption and photocatalysis. Efficient adsorption was observed under typical wastewater pH conditions, with a maximum adsorption capacity of 23.4 mg·g^−1^, which fit well with the Langmuir isotherm model. The key mechanisms governing MB adsorption were identified as ion exchange, electrostatic attraction, and hydrogen bonding. The adsorption process was exothermic, with kinetic data fitting both the pseudo-second order and intraparticle diffusion models, achieving 82% removal and a maximum adsorption capacity of 40 mg·g^−1^ over 12 h. MB adsorption followed a two-step process, initially involving film diffusion, followed by intraparticle diffusion. Additionally, photocatalytic degradation of MB achieved 77% degradation within 180 min. However, a decrease in reusability was observed during a second cycle of MB adsorption and photodegradation, highlighting the need for further optimization to enhance the material’s long-term performance.

## 1. Introduction

The textile industry significantly contributes to the economic development of many countries; however, it is also a major cause of water pollution due to the large amounts of wastewater discharged [1]. Textile dyeing industries release wastewater containing harmful organic compounds, such as dyes, which are non-degradable and highly carcinogenic with serious risks to human health [2]. These dyes also increase water turbidity, which inhibits the absorption of dissolved oxygen by aquatic organisms. Additionally, their bioaccumulation and biomagnification in the aquatic environment pose significant risks to human health, particularly through the consumption of contaminated seafood [3]. Therefore, removing dyes from textile wastewater is a public health priority, and countries must strictly regulate dye discharges to protect natural water bodies in accordance with mandatory safety levels [4].

Methylene blue (MB) is a heterocyclic aromatic dye containing sulphur, classified as an azo dye based on its chemical structure. MB is conventionally used in the textile industry to dye cotton, silk, and wool [5]. However, exposure to MB can cause undesirable health effects, including breathing difficulties when inhaled, and nausea, diarrhoea, vomiting, itching, and gastritis when ingested. These effects are particularly severe at high concentrations of MB [6]. Several methods have been explored for the removal of organic pollutants, such as adsorption, ion exchange, photocatalysis, chemical oxidation, and coagulation [7]. Among these, catalytic degradation and adsorption have gained significant attention in recent years due to their effectiveness in removing harmful contaminants from water. Several materials have been used for MB adsorption, including activated carbon [8], magnetic zeolites from recycled fly ash [9], and zeolites modified with magnetic graphene oxide [10].

Adsorption is one of the most widely used methods for pollutant removal due to its high efficiency and ease of operation [11]. It is also economically advantageous compared to other technologies and is practical for removing non-degradable pollutants such as methylene blue. Chemical and physical adsorption, as well as ion exchange, have been reported as the main mechanisms involved in adsorption processes [11]. However, developing efficient adsorbent materials with high adsorption capacities remains a challenge [12]. Recent studies have focused on obtaining low-cost adsorbents with enhanced adsorption efficiency, using natural minerals, agricultural wastes, and industrial wastes [13]. In this context, zeolites have been extensively studied as natural minerals for adsorption applications, with several synthesized types of zeolites used for this purpose.

Naturally occurring zeolites are often contaminated with other minerals, metals, quartz, and other zeolitic phases, which limits their purity and, consequently, their commercial applications [14]. However, zeolites can also be synthesized using different methods and various raw materials. For industrial purposes, high purity and a consistent chemical composition are essential. Therefore, the hydrothermal method, under conditions of high temperature or pressure and using natural raw materials and synthetic silicates, is preferred [15]. Synthetic zeolites are typically obtained from pure reagents such as sodium aluminate, sodium silicate, and sodium hydroxide, at specific molar ratios (SiO_2_/Al_2_O_3_; Na_2_O/SiO_2_ and H_2_O/Na_2_O). The use of natural aluminosilicate sources (such as clays and diatomaceous earth) has also been explored, though these require additional treatments such as thermal activation and acid treatment [16]. In fact, some zeolites have been synthesized from industrial waste, including materials from feldspar processing [17], fly ash [18] and mine tailings [19], offering an environmentally sustainable and cost-effective alternative to commercially available zeolites.

The synthesis of zeolites from industrial wastes has advantages compared to commercial zeolites synthesized from pure chemicals. These zeolites are cheaper than commercial ones, and the use of industrial wastes as sources of Al and/or Si contributes to environmental sustainability by enabling waste reuse and recycling [20]. The production of zeolites from waste offers a value-added product with unique physical and chemical properties, suitable for various practical applications, including adsorption and catalysis. Zeolites are hydrated and crystalline aluminosilicates, and their structure consists of silica and alumina tetrahedrons linked by shared oxygen atoms, containing well-defined channels and chambers filled with ions and water molecules [21]. Zeolites are excellent cation exchangers, making them optimal for the removal of cationic pollutants like methylene blue.

Incorporating metals species into the zeolite structure can modify its structural and acidic properties, further improving its ion-exchange and adsorption capacity [22]. Cation exchange in zeolites enable the pores to be effectively tuned for selectivity, allowing easier diffusion and adsorption of adsorbate molecules [23]. However, the use of powdered adsorbents can be challenging for large-scale applications due to blockages in pipes and the potential for secondary contamination. Consequently, the synthesis method plays a crucial role in determining the physicochemical characteristics, cost, and overall performance of the synthesized materials [24]. In our previous study, we evaluate the experimental conditions for synthesizing LTA zeolites from mining tailings [25]. In this study, we synthesized a highly crystalline LTA zeolite and exchanged it with lithium ions, resulting in a novel material with significantly enhanced methylene blue adsorption properties. This low-cost material exhibits improved adsorption properties towards MB, and to the best of our knowledge, no previous studies have extensively detailed the physicochemical properties and adsorption mechanisms of this novel material.

Lithium hydroxide (LiOH) has been successfully used in several catalytic applications. Many examples report the enhancement of photocatalytic activity due to LiOH, including increased specific surface area and improved charge separation efficiency of graphitic carbon nitride [26], a robust catalytic system LiOH–FeNiO*_x_* for an aldol reaction converting ethanol to butanol [27], a LiOH-pumice catalyst for the production of biodiesel from lyophilized *Chlorella* sp. [28], and LiOH in a 3 wt% LiOH-promoted, α-Al2O3-supported 1 wt% Ru catalyst for NH_3_ decomposition in H_2_ production [29]. In this study, we aimed to synthesize LiOH on the surface of MT-ZLSH and evaluate its potential for the photocatalytic degradation of MB.

The catalyst synthesis method is crucial for determining effectiveness, cost, particle size, morphology, and active phase dispersion. Achieving an appropriate distribution of active species is essential for exposing more active sites, which enhances catalytic performance [30]. While microwave catalysis has emerged as a promising technology for water treatment, offering improved stability and performance [31], its high energy requirements limit its scalability. Thus, low-cost synthesis methods for catalytic applications are highly desirable. Incorporating lithium into zeolite structures offers significant advantages in enhancing adsorption and photocatalytic performance. However, as lithium is a high-cost, strategic resource, there is growing interest in finding more sustainable sources of lithium. In this regard, lithium-containing waste from spent lithium-ion batteries presents a promising alternative. Using such waste not only reduces costs due to the high availability of these materials but also contributes to a circular economy by promoting lithium recycling.

LiOH is conventionally synthesized using lithium carbonate (Li_2_CO_3_) as a precursor due to its low solubility in water (1.54 g/100 g at 0 °C), obtaining a conversion into LiOH above 50% with the co-existence of both Li_2_CO_3_ and LiOH phases [32]. The synthesis of LiOH has also been reported via the precipitation method from recycled lithium sulphate (Li_2_SO_4_) from discarded Li-containing products. The use of Li_2_SO_4_, which has high solubility in water (26.1 g/100 g at 0 °C), was successful achieved through a two-step precipitation method [33]. The synthesis of lithium hydroxide from lithium chloride (LiCl; 68.29 g/100 g at 0 °C) and sodium hydroxide (NaOH; 41.8 g/100 g at 0 °C) as precursors, both highly soluble in water, has not been extensively explored. Thus, in this study, we propose the synthesis of LiOH using these precursors, supported on MT-ZLSH obtained from mining tailings. Although we used pure lithium chemicals in this initial phase of research, future studies can be focused on using lithium from battery waste to make the process more cost-effective and environmentally sustainable.

This study aims to evaluate lithium zeolite (MT-ZLSH-Li^+^) synthesized hydrothermally from mining tailings for methylene blue adsorption and photocatalytic activity. The primary objectives are to (i) evaluate the effect of pH on methylene blue removal, (ii) determine kinetic parameters, (iii) establish the maximum adsorption capacity, (iv) investigate thermodynamic behaviour, and (v) evaluate the photocatalytic activity of MT-ZLSH-Li^+^. Given the relevance of methylene blue removal, understanding these processes is crucial for developing industrial wastewater treatment technologies, which can be readily scaled for laboratory and industrial applications.

## 2. Results and Discussion

### 2.1. Physicochemical Characterization of MT-ZLSH and MT-ZLSH-Li^+^

The chemical composition of the synthesized MT-ZLSH and the lithium-modified zeolite MT-ZLSH-Li^+^ is presented in Table 1. Alumina (Al_2_O_3_) and silica (SiO_2_) were identified as the major components of these zeolitic materials, forming a framework of alternating aluminium and silicon oxide of tetrahedrons that share corners, creating a cubic structure consisting of eight alpha cages [34]. Both materials displayed a Si/Al ratio close to one, which is typical for this type of zeolite and suggest a high cation exchange capacity [35]. The chemical composition of both zeolitic materials remained unchanged, indicating that the incorporation of lithium did not alter the structure of the synthesized MT-ZLSH [23]. Chloride was not detected in the MTA-ZLSH-Li^+^ samples via FE-SEM. For lithium quantification, inductively coupled plasma optical emission spectroscopy was employed, as Energy Dispersive Spectroscopy (EDS) is unsuitable for identifying and quantifying light elements like lithium, which have an atomic number less than six.

The ion exchange reaction between Li^+^ and ions from the ZLSH zeolite framework was confirmed by measuring the released elements in the exhausted solution: Na, Fe, Ca, K, and Zn (via atomic absorption) and Li, Si, and Al (via inductively coupled plasma optical emission spectroscopy). The incorporation of lithium into ZLSH zeolite was found to be 4.41 mg·g^−1^. The relatively low amount of Li^+^ dopped can be attributed to the preparation method employed in this study, which involved the high solubility of lithium hydroxide in water (12.7 g/100 mL at 0 °C). A significant portion of LiOH was lost during preparation (specifically during the washing of the lithium zeolite), where the primary objective was to remove sodium chloride, which has even higher solubility (35.7 g/100 mL at 0 °C). To quantify the lithium supported on the zeolite, the difference was calculated between the initial lithium content of the LiCl solution and the remaining lithium in the exhausted LiCl solution, accounting for the lithium lost during the washing of the zeolite. Despite the high solubility of lithium hydroxide, it was assumed that not all supported LiOH was lost during washing. However, further tests are needed to corroborate this assumption. The production of lithium zeolite followed the previous reported behaviour, where Li^+^ ions exchanged with Na^+^ ions [36]. The incorporation of lithium led to the released of sodium as the main exchanged species (18 mg·L^−1^, attributed to the NaOH used in the synthesis of MT-ZLSH-Li^+^), compared to aluminium (10 mg·L^−1^), potassium (2 mg·L^−1^), silicon (2 mg·L^−1^), zinc (1 mg·L^−1^), calcium (0.3 mg·L^−1^), and iron (0.1 mg·L^−1^), which were present in lower concentrations in the exhausted solution. The ion exchange reaction between the zeolite framework and lithium was mainly constrained by the ionic radius [37] with monovalent lithium (90 pm) easily occupying sites on ZLSH zeolite vacated by sodium (116 pm) > potassium (152 pm) > zinc (74 pm) > calcium (114 pm) > iron (65 pm) [25].

**Figure 1:**
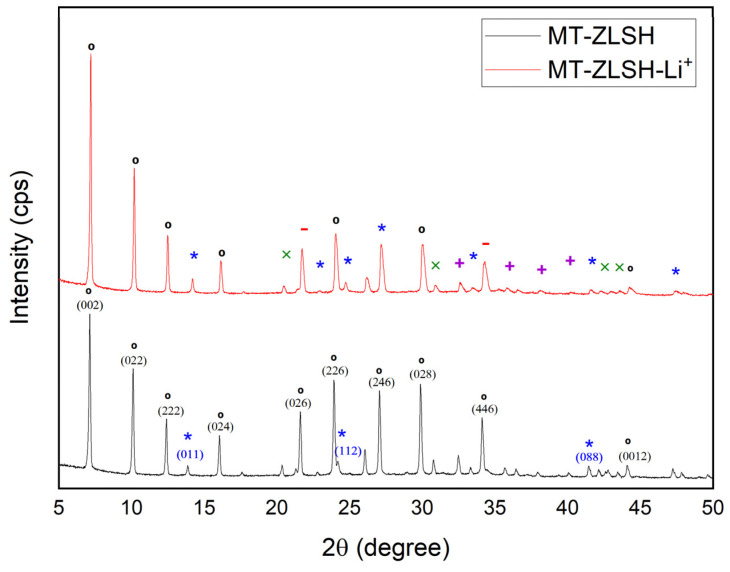
XRD patterns of the synthesized zeolite MT-ZLSH and MT-ZLSH-Li^+^. * Sodalite and ° LTA zeolitic phases, ^−^ Cancrinite, ^x^ LiOH, and ^+^ Li_2_CO_3_.

The X-ray diffraction (XRD) patterns of MT-ZLSH and MT-ZLSH-Li^+^ are shown in Figure 1. Characteristic reflections of LTA, sodalite–hydroxysodalite, and cancrinite were consistent with those of the International Zeolite Association [38]. LTA peaks were indexed to the cubic crystal system and space group Pm-3m, with refined unit cell parameters a[Å] = b[Å] = c[Å] = 11.96. The sodalite peaks were indexed to the cubic crystal system and the space group P-43n, with refined unit cell parameters a[Å] = b[Å] = c[Å] = 8.98. Hydroxysodalite has nearly similar crystallography as its parent zeolite, sodalite, while the cancrinite peaks were indexed to the hexagonal crystal system and the space group P 63/m mc, with refined unit cell parameters a[Å] = b[Å] = c[Å] = 12.5. In MT-ZLSH, the main reflections for LTA zeolite were observed at 2θ: 7.1° (100), 10.1° (110), 12.4° (111), 16.0° (210), 22.8° (300), 23.4° (310), 27° (320), 29.8° (400), 34.1° (421), and 44.0° (530). Sodalite reflections were observed at 2θ: 13.9° (110); 20.3° (200), 24.2° (211), 28.9° (220), 37.9° (321), 42.1° (411), and 47.8° (332), while cancrinite reflections were observed at 2θ: 21.7° (210) and 35.9° (320). The diffraction pattern of MT-ZLSH-Li^+^ exhibited slight changes compared to MT-ZLSH, suggesting that the incorporation of lithium affected the crystallographic parameters without compromising the overall structure. In MT-ZLSH-Li^+^, the sodalite reflection at 2θ: 41.4° (088) disappeared, and the intensity of LTA reflections at 2θ: 29.8° (028) and 34.1° (466) decreased. Changes in the crystallographic parameters of MT-ZLSH and MT-ZLSH-Li^+^ were measured, with unit cell parameters of LTA and sodalite in MT-ZLSH-Li^+^, adjusting to a[Å] = b[Å] = c[Å] = 11.91 and 9.06, respectively. The intensity of reflections between MT-ZLSH-Li^+^ and MT-ZLSH changed, consistent with reports for lithium-exchanged zeolites. However, the quantitative XRD analysis indicated an increase in sodalite–hydroxysodalite and cancrinite contents without deterioration of the structure following lithium incorporation. This can be explained by the ion exchange process, where cations occupy the pores, and their scattering power is specific to each species [37]. When zeolite 13X was impregnated with LiCl and CaCl_2_, new peaks appeared between 30° and 35°, and the intensity of peaks increased as the LiCl:CaCl_2_ salt ratios increased [39]. In contrast, the decrease in reflections observed in MT-ZLSH-Li^+^ compared to parent MT-ZLSH was notable. Reflections in MT-ZLSH-Li^+^ also indicated the formation of ≤1.6% lithium hydroxide and lithium carbonate, present in small quantities that were compared with standard patterns. The diffraction peaks at 20°, 22°, 30°, 32°, 33°, 34°, 37°, 38°, 40°, 41°, 43°, 49°, and 51° were commonly attributed to LiOH·H_2_O [40]. Similarly, diffraction peaks at 22°, 24°, 29°, 31°, 32°, 34°, 36°, 38°, 39°, 40°, 43°, 44°, and 49° were attributed to Li_2_CO_3_ [32]. Some of these characteristic reflection peaks, as reported in the literature for LiOH·H_2_O and Li_2_CO_3_ were found in MT-ZLSH-Li^+^, but with minor changes such as lower intensity and broader peaks. A similar effect was reported for carbon nanostructures when LiOH·H_2_O was supported on them, leading to broad diffraction peaks indicating their dispersion across the nanostructures [40]. The presence of Li_2_CO_3_ can be explained by the partial conversion of LiOH into Li_2_CO_3_ when heated above 100 °C in ambient air, as was undertaken in this study at temperatures exceeding 200 °C [41]. This is consistent with the coexistence of LiOH and Li_2_CO_3_ species, as previously reported [33].

The parent MT-ZLSH exhibited a d-spacing for LTA zeolite of 12.40 Å at 2θ: 7.12 (100), whereas the MT-ZLSH-Li^+^ zeolite showed a d-spacing of 12.30 Å at 2θ: 7.19 (100), suggesting a structural rearrangement of the LTA due to lithium incorporation via ion exchange. Additionally, the content of hydroxysodalite and cancrinite zeolite phases increased, along with the incorporation of small amounts of lithium hydroxide and lithium carbonate into MT-ZLSH-Li^+^.

The detection of aluminium and silicon in the exhausted solution following Li^+^ incorporation suggests that Li^+^ was incorporated through the removal of some framework aluminium and the substitution of extra-framework Al^3+^ species. This likely promoted the substitution of tetrahedral framework Si^4+^ species with Al^3+^, leading to the formation of some extra-framework species. Another potential mechanism for Li^+^ incorporation into the zeolite framework is electrostatic attraction, facilitated by the natural negative charge of zeolites [37].

In this study, the use of NaOH to increase the pH to 12 during the incorporation of Li^+^ into MT-ZLSH promoted the formation of hydroxysodalite and cancrinite—a process that has been previously reported when NaOH and CaCO_3_ are incorporated into in the crystal structure of sodalite [42]. The increase in both zeolitic phases (hydroxysodalite and cancrinite) was clearly observed in scanning electron microscope (SEM) micrographs, which revealed multiple nanostructures supported over LTA zeolite cubes and lephispheres. The SEM analysis also revealed the existence of a thin, sparsely dispersed film of lithium hydroxide and lithium carbonate, which contribute to the altered morphology of MT-ZLSH-Li^+^. Experimental conditions indicated the coexistence of metal hydroxides, such as LiOH, along with their ionic species Li^+^ and OH^−^. Thus, Li^+^ was found to be incorporated into MT-ZLSH-Li^+^ through ionic exchange and electrostatic attraction, with precipitation of LiOH to a lesser extent. There results align with the distribution diagram of the logarithmic concentration of Li^+^ species as a function of pH for the LiCl (0.1 M Li^+^) system, as previously reported [25].

**Figure 2:**
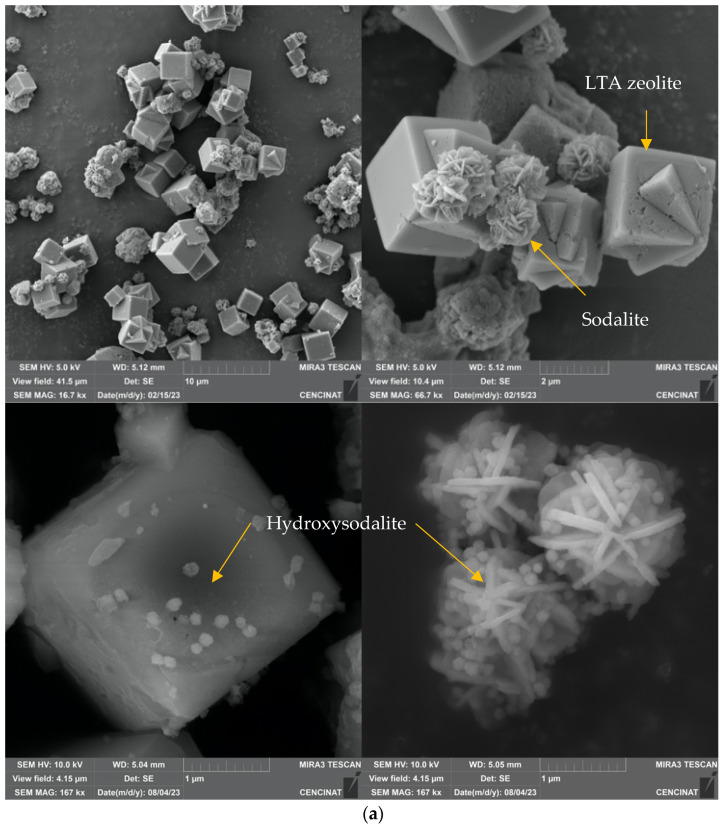

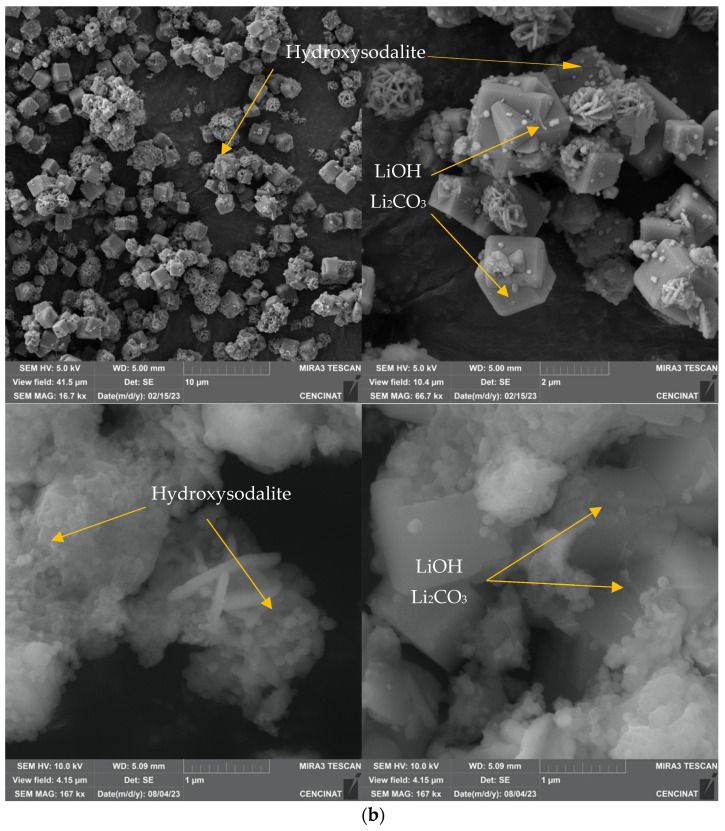
SEM micrographs (scale bar from 1 to 10 μm) of (**a**) MT-ZLSH and (**b**) MT-ZLSH-Li^+^.

Figure 2 depicts the morphology of both MT-ZLSH and MT-ZLSH-Li^+^. The morphology of the MT-ZLSH zeolite primarily exhibited characteristic features of LTA and sodalite zeolites, including a combination of well-defined cubes and lephispheres (Figure 2a) [43,44]. In certain regions of MT-ZLSH, spherical hydroxysodalite nanoparticles, which belong to the sodalite family, were observed to be sparsely dispersed across the cube faces of LTA zeolite and between the sodalite lephispheres (Figure 2a). After the treatment of parent MT-ZLSH, areas with a higher concentration of hydroxysodalite zeolite species were found on the surface of synthesized MT-ZLSH-Li^+^. Additionally, a thin film of sparsely dispersed particles was detected in specific regions of MT-ZLSH-Li^+^, which were attributed to lithium hydroxide and lithium carbonate nanoparticles. This thin particle film covered some areas of the sphere-shaped hydroxysodalite particles, the cube faces of LTA, and the surface of sodalite lephispheres (Figure 2b). The limited presence of this thin layer of lithium nanoparticles aligns with the small amount of lithium detected in analytical measurements, likely due to losses during the washing process. The morphology of lithium hydroxide and lithium carbonate incorporated into the MT-ZLSH-Li^+^ resembles that of LiOH dispersed on the surface of a porous carbon composite [45]. These lithium hydroxide nanoparticles also exhibited an irregular morphology similar to the nanorods synthesized from the Li_2_SO_4_ precursor [33]. However, the typically globular nano-scale features associated with lithium carbonate [33] were not readily visible in the SEM micrographs from this study. The crystal structure and morphology of the synthesized LiOH are primarily influenced by the solubility of the precursor, which plays a key role in the nucleation and growth mechanism [32]. Interestingly, the morphology of MT-ZLSH-Li^+^ differed from a lithium-exchanged LTA zeolite [37], where no significant surface changes were observed after lithium incorporation, resulting in a smooth surface. In our study, the particle size of LTA zeolite in MT-ZLSH ranged between 1.5 and 2.5 µm, while smaller particles of sodalite, around 1.3–1.4 µm were noted. The size of hydroxysodalite particles ranged between 50 nm and 100 nm. The particle size of lithium hydroxide and lithium carbonate, which are rarely found on the surface of MT-ZLSH-Li^+^, exceeded 25 nm. As mentioned earlier, the particle size, morphology, and particle density of lithium hydroxide depend on the selected precursor, reaction time, precursor ratios, and evaporation temperature [32,33,41,45].

**Figure 3:**
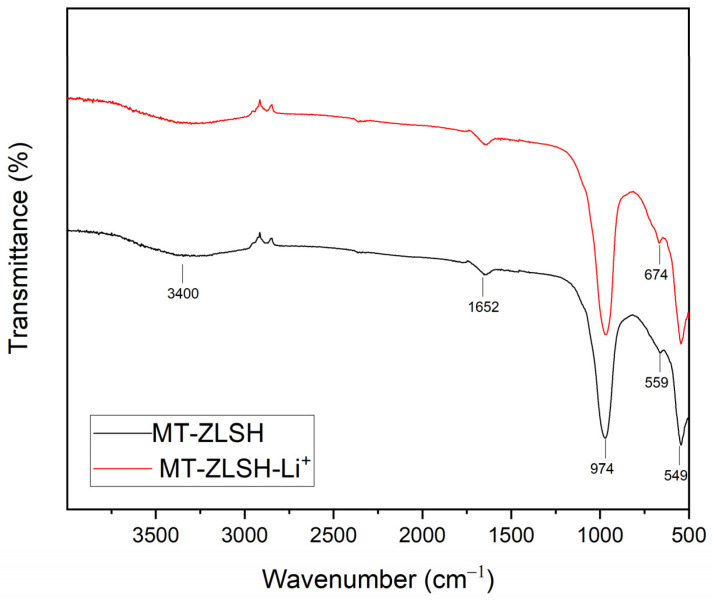
FTIR spectra of MT-ZLSH and MT-ZLSH-Li^+^.

The Fourier transform infrared spectroscopy (FTIR) spectrum of both MT-ZLSH and MT-ZLSH-Li^+^ is presented in Figure 3. The main peaks were observed at 974 cm^−1^ corresponding to the asymmetric stretching vibration of the internal TO_4_ tetrahedron. The band at 658 cm^−1^ was attributed to the symmetric vibration of the double ring in TO_4_ tetrahedrons. The peak at 549 cm^−1^ represented the secondary building unit in the zeolite LTA (D_4_R). Characteristic bands associated with the zeolite structure were identified in the MT-ZLSH sample [46]. The band at 3400 cm^−1^ was ascribed to both intramolecular and intermolecular hydrogen bonding and intertwined interstitial water [36]. The peak at 1640 cm^−1^ was attributed to the bending vibration of H–O–H [47]. Bands between 733 and 660 cm^−1^ were linked to the presence of sodalite, consistent with previous studies [48]. In the MT-ZLSH-Li^+^ spectrum, the intensity of the band at 676 cm^−1^ changed, which was attributed to the symmetric vibration of the double ring in TO_4_ tetrahedrons. Changes in the region between 400 and 700 cm^−1^ were recognized as indicators of cation movement, possibly due to the interaction between water and Li^+^, leading to distortion [49]. This is associated with the incorporation of Li^+^ via ionic exchange and electrostatic attraction as the primary mechanisms. Additional changes were observed in the OH groups region around 3310 cm^−1^, corresponding to hydroxyl groups (mainly Al-OH and Si-OH) that are present in zeolites (LTA, sodalite and hydroxysodalite) [50]. These changes resulted from the transformation of sodalite into hydroxysodalite and cancrinite, which was accompanied by the precipitation of small amounts of lithium hydroxide and lithium carbonate on the zeolite surface [33]. Moreover, changes around the peak at 974 cm^−1^ were attributed to the conversion of sodalite into hydroxysodalite and cancrinite—a phenomenon previously associated with zeolite formation [42,51]. Slight shifts in the FTIR absorption bands were observed when lithium was incorporated into the MT-ZLSH zeolite, and these were compared with the patterns of lithium hydroxide and lithium carbonate. The minor shifts in absorption bands between 2800 cm^−1^ and 2900 cm^−1^, as well as around the 3400 cm^−1^ peak, could be associated with lithium hydroxide, while the absorption bands at 1400 cm^−1^ and 2300 cm^−1^ were associated with lithium carbonate. This effect can be attributed to the low content of these lithium compounds supported on the surface of MT-ZLSH-Li^+^.

### 2.2. Effect of pH on Adsorption

**Figure 4:**
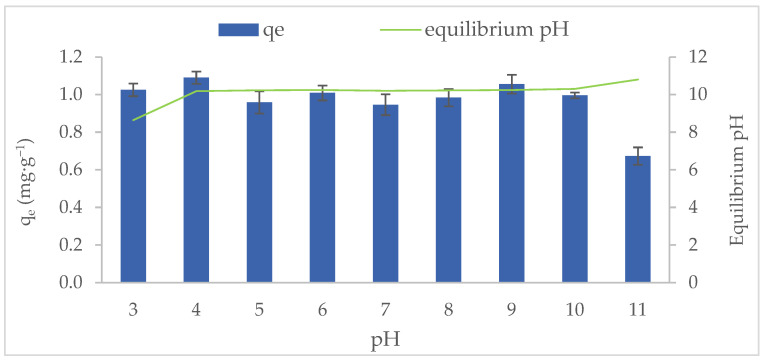
Methylene blue adsorption onto MT-ZLSH-Li^+^ as a function of the initial and equilibrium pH.

The MB adsorption capacity of MT-ZLSH-Li^+^ as a function of solution pH is showed in Figure 4. The adsorption was evaluated across a pH range of 3 to 11, remaining stable under most conditions, except at pH 11. The adsorption behaviour of MT-ZLSH-Li^+^ towards MB can be explained by three main factors: (1) the surface charge of the adsorbent; (2) the degree of ionization of the adsorptive molecule; and (3) the dissociation of functional groups on the active sites of the adsorbent [52]. The dissociation of specific functional groups on the active sites can be attributed to M-OH groups (M: Al, Na, K, Zn, Ca, Fe and Li) present on the MT-ZLSH-Li^+^ zeolite. The activity of zeolites is largely determined by their crystalline aluminosilicate structure, which creates Brønsted acid sites (BAS), while defects may introduce Lewis acid sites (LAS). Brønsted acidity in zeolites arises from hydroxyl groups that bridge framework silicon and aluminium atoms. In contrast, the structure of Lewis acid sites is less well-defined but serves as active surface sites, functioning as electron pair acceptors. As a result, numerous studies have focused on developing Lewis acid zeolites to improve their adsorption and catalytic potential [53,54]. Lewis acidity in zeolites can be achieved by incorporating heteroatoms into the framework or by post-synthesis treatments that remove some framework aluminium, leading to the formation of extra-framework species, such as the simple ion-exchange of aluminium species, as performed in this study [54]. Traditionally, extra-framework aluminium species in zeolites have included Al^3+^, Al-(OH)^2+^, Al(OH)^2+^, AlOOH, Al(OH)_3_, and Al_2_O_3_. However, only the cationic extra-framework species, Al^3+^, Al(OH)^2+^, and Al(OH)^2+^ are considered strong Lewis acid sites [53,54]. Consequently, changes in the distribution of framework aluminium atoms affect the acid strength of the hydroxyl groups in zeolite, as seen with the Al-OH groups after Li^+^ incorporation [53]. Moreover, the silanol Si-OH hydroxyl groups present in zeolites, although considered structural defects, play a significant role in influencing overall acidity and, consequently, the final properties and applications of zeolites. The acidity of silanol hydroxyls cannot always be fully determined by the spectral characteristics of zeolites but substantially contributes to hydrogen bond formation and strength, which depends on their configuration within the zeolite structure. Silanol groups have demonstrated sufficient acidity for catalytic application where such acidic sites are critical, even in the absence of other strong acid sites [55]. Consistent with previous reports, the M-OH groups (Al-OH and Si-OH) in zeolites (LTA, sodalite—hydroxysodalite and cancrinite) are primarily responsible for MB adsorption. Hydration reactions promote the formation of OH or OH^2+^ groups, as shown in Equations (1)–(3) [56]. Thus, ion exchange reactions play a critical role in the adsorption of MB onto MT-ZLSH-Li^+^ under acid conditions [57]. In this study, lithium hydroxide dissociated into Li^+^ and OH^−^, further contributing to ion exchange reactions and increasing the number of active sites on the adsorbent’s surface.

(1) $$MT-ZLSH-M-OH+MB^{+}↔MT-ZLSH-M-O-MB+H^{+}$$

(2) $$MT-ZLSH-M-OH+H^{+}↔MT-ZLSH-M-OH_{2}^{+}$$

(3) $$MT-ZLSH-M-OH_{2}^{+}+2MB^{+}↔MT-ZLSH-M-O-MB_{2}^{+}+2H^{+}$$

On the other hand, the deprotonation reaction show in Equation (4) can lead to the formation of functional M-O^−^ groups at higher pH levels, which promotes MB adsorption through an electrostatic interaction as described in Equation (5) [56]:

(4) $$MT-ZLSH-M-OH↔MT-ZLSH-M-O^{-}+H^{+}$$

(5) $$MT-ZLSH-M-O^{-}+MB^{+}↔MT-ZLSH-M-O-MB$$

The increase in pH values is due to the acid-base neutralization reaction, as depicted in Equation (6), which favours the deprotonation reaction Equations (4) and (5) while inhibiting hydration reactions Equations (1)–(3). This observation aligns with the experimental data, where equilibrium pH values were between 8.6 and 10.8. As a result, the concentration of M–O^−^ increases, while the concentration of M–OH^2+^ decreases [56].

(6) $$H^{+}-OH^{-}↔H_{2}O$$

The surface charge of MT-ZLSH-Li^+^ was determined by the point of zero charge, pH_pcz_ = 9.5 ± 0.1. The surface of MT-ZLSH-Li^+^ is positively charged below the pH_pcz_ and negatively charged above the pH_pcz_ [58]. Additionally, the degree of ionization of the MB molecule is estimated based on its reported pK_a_= 3.8 [52]. Thus, when the pH is below 3.8, the predominant form of the MB molecule in solution carries a positive charge. At a pH of 3.8, the dominant species is a neutral amphiphilic MB molecule. Finally, when the pH is above 3.8, the predominant MB molecule in solution carries a negative charge [59].

Below the pH_pcz_, the ion exchange reaction explains the adsorption of MB onto the positively charged MT-ZLSH-Li^+^ surface [60]. However, the positively charged MT-ZLSH-Li^+^ surface can also interact with negatively charged species of the MB molecule (present above pK_a_= 3.8) through electrostatic attraction. Above the pH_pcz_, the negatively charged surface of MT-ZLSH-Li^+^ promotes the formation of M–O^−^ groups, which electrostatically attract the positively charged MB molecules [60]. The slight reduction in MB adsorption capacity at pH values much higher than the pH_pcz_ suggest the involvement of an additional mechanism. The presence of M–O^−^ bonding sites on the MT-ZLSH-Li^+^ surface restricts the adsorption of some negatively charged species of the MB molecule (above pK_a_ = 3.8). The results of this study indicate that both ion exchange and electrostatic interactions are the primary mechanism governing MB adsorption onto MT-ZLSH-Li^+^.

### 2.3. Adsorption Isotherms

**Figure 5:**
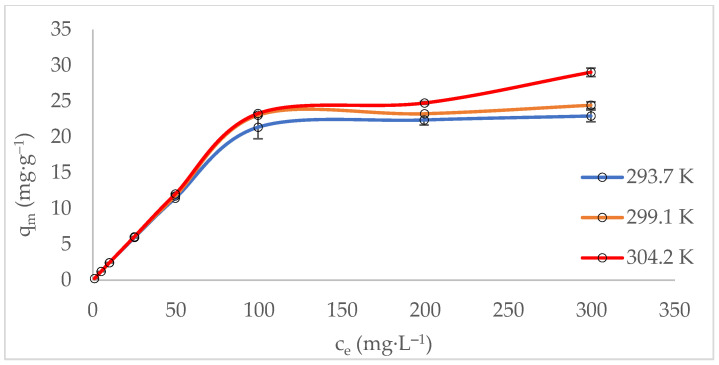
Equilibrium adsorption data: isotherms as a function of temperature.

The adsorption isotherm describes the equilibrium behaviour of the zeolite (adsorbent) at a constant temperature. It depends on several factors, including the adsorbate (methylene blue), the adsorbent (zeolite), and the physical properties of the solution, such as pH, ionic strength, and temperature [61]. The experimental data were fitted to both Langmuir and Freundlich isotherm models (Table 2). The adsorption of MB was best described by the Langmuir isotherm model, suggesting a chemisorption mechanism. This indicates that the surface of MT-ZLSH-Li^+^ was homogeneous, with adsorption sites of equal energy and monolayer adsorption capacity. The maximum adsorption capacity of methylene blue onto MT-ZLSH-Li^+^ was 23.4 mg·g^−1^, which is higher than that reported for other adsorbent materials [9,62]. According to the Freundlich isotherm model, the correlation coefficient ranged between 0.72 < R^2^ < 0.78, indicating that some degree of heterogeneous adsorption sites and multilayer physical adsorption occurred, though to a lesser extent [56]. At the evaluated temperatures, the experimental data aligned more closely with the Langmuir isotherm model, with separation factor ($r_{L}$) values between 0 < $r_{L}$ < 1, demonstrating that the MB adsorption onto MT-ZLSH-Li^+^ was favourable [63]. On the other hand, the Freundlich parameters (1/n) had values in the range of 0.49–0.50, also suggesting the experimental data were reasonably described by the Freundlich model [62]. The adsorption capacity of MB increased with temperature, rising from 23.4 mg·g^−1^ at 293.7 to 28.8 mg·g^−1^ at 304.2 K (Figure 5).

### 2.4. Adsorption Thermodynamics

The thermodynamic parameters provide insights into the adsorption mechanisms between methylene blue and MT-ZLSH-Li^+^. These parameters are detailed in Table 3. The ΔG^0^ values were negative and decreased with increasing temperature, indicating that the adsorption process was spontaneous and more favourable at higher temperatures, with greater retention of adsorbed MB. The positive values of ΔS^0^ $\Delta S^{0}$ suggest a dissociative adsorption mechanism, promoting an increase in the degree of freedom at the liquid-solid interface [64]. The negative ΔH^0^ value confirms that the process was exothermic, meaning energy was released during adsorption. Furthermore, the small magnitude of ΔH^0^ implies that the adsorption process was primarily governed by physisorption [65]. The increase in MB adsorption capacity with temperature may be attributed to the enhanced interaction between MB molecules and the active sites on the zeolite. The adsorption process likely began with the external transfer of MB to the surface, followed by diffusion into the internal pores of the adsorbent material. As temperature increased, the viscosity of the MB solution decreased, thereby improving MB mobility [57]. Additionally, higher temperatures promote stronger chemical interactions due to the increase in available adsorption sites. This may be caused by the destruction of internal bonds near the edges of the active surface sites of MT-ZLSH-Li^+^, the creation of new active sites, or changes in chemical potentials associated with MB solubility [66].

### 2.5. Kinetic of Adsorption

**Figure 6:**
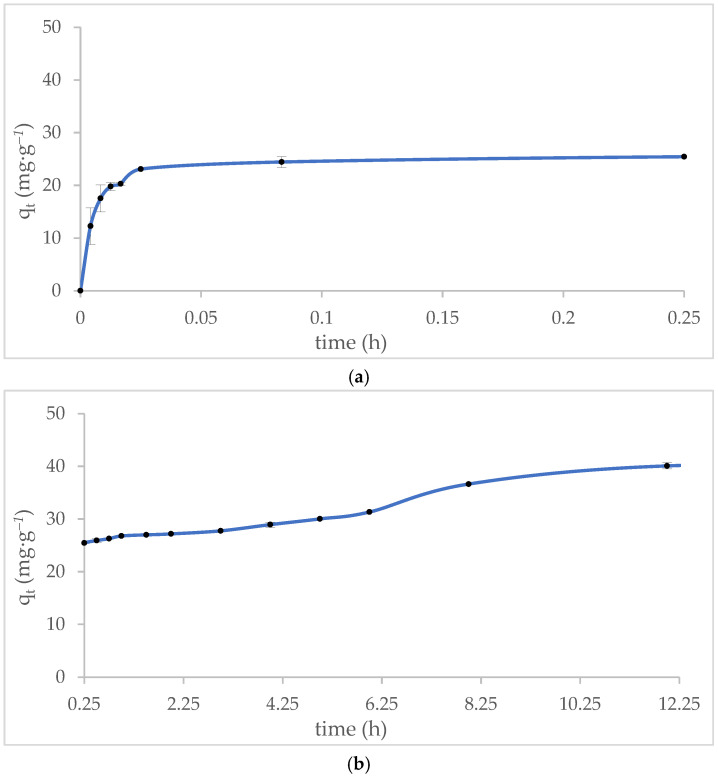
Effect of contact time between the adsorbent and methylene blue: (**a**) initial adsorption behaviour during rapid uptake (within the first 15 min); (**b**) adsorption behaviour over time (between 15 min and 12 h).

The methylene blue (MB) adsorption profile on MT-ZLSH-Li^+^ is depicted in Figure 6. Approximately 50% of MB was removed within the first 5 min of contact (Figure 6a), and 67% removal was achieved after one hour. Following this, the adsorption rate slowed, reaching around 70% MB removal within 5 h. The rate of adsorption then decreased further until equilibrium was reached, resulting in a notable 87% removal of MB after 12 h, with a final adsorption capacity of q_t_ = 40 mg·g^−1^ (Figure 6b). The results of this study are not directly comparable to those of other lithium zeolites due to the lack of similar studies. However, the MB adsorption onto MT-ZLSH-Li^+^ is consistent with reports on other zeolite composites, which required approximately 8 h to reach equilibrium [58]. This is similar to a synthesized zeolite using aluminium isopropoxide, which reached equilibrium within 5 h. Conversely, a zeolite synthesized from pure sodium aluminate reagent achieved 83.28% MB removal within just 60 min [67]. The adsorption behaviour of MB appears to depend on the zeolitic phase, which is influenced by the chemical, physical, and crystallographic properties of the material, the precursors used, and the specific mechanisms involved between MB and the adsorbent [52]. For instance, a study conducted using iron mine waste from Brazil reached MB adsorption equilibrium in 200 min, with 45% MB removal. However, that study used a higher concentration of MB and a larger amount of adsorbent [68]. The adsorbent MT-ZLSH-Li^+^ used in this study demonstrate improved properties compared to the previously mentioned materials, as it required only a small amount of adsorbent (0.1 g).

The methylene blue (MB) adsorption data were fitted to the kinetic models described in Table 4. The adsorption of MB onto MT-ZLSH-Li^+^ was best described by the pseudo-second order kinetic model, with R^2^ = 0.98. Additionally, the calculated q_e_ value for MB adsorption showed excellent agreement with the experimental q_e_ value. In the case of natural zeolites, the kinetics of MB adsorption have previously been attributed to chemisorption—a process explained by the sharing or exchange electrons between the adsorbent and the adsorbate, consistent with valency forces [52,60]. Chemical adsorption has also been reported in previous studies on iron mine waste, further establishing chemisorption as the principal mechanism of MB adsorption [68].

The experimental data was also well described by the intraparticle diffusion model. The plot of the intraparticle diffusion kinetic model indicates that there was not a single rate-limiting step during MB adsorption, revealing the existence of multi-stage adsorption. Four distinct stages were identified for MB adsorption onto MT-ZLSH-Li^+^. In the first stage, ultra-fast adsorption of methylene blue occurred, with the highest kinetic constant value of k_t1_ = 361.05 mg·g^−1^. The second stage was characterized by a slower phase with k_t2_ = 114.13 mg·g^−1^. The third stage showed the lowest kinetic constant value of k_t3_ = 1.64 mg·g^−1^. Finally, the fourth stage was marked by the equilibrium phase, with a kinetic constant of k_t4_ = 2.93 mg·g^−1^. Our results suggest that the influence of film or pore diffusion, or a combination of both, governed MB adsorption onto MT-ZLSH-Li^+^—a process that is highly dependent on the system’s hydrodynamics [52]. In the first stage, MB adsorption occurred via film diffusion through the hydrodynamic layer. The second stage involved diffusion through the boundary layer to the external surface of the MT-ZLSH-Li^+^ zeolite. In the third stage, the sharp reduction in the adsorption rate was driven by intraparticle diffusion within the porous structure of MT-ZLSH-Li^+^. Finally, in the fourth stage, the adsorption process was characterized by decreasing MB concentration in the solution and the reduction of active sites on MT-ZLSH-Li^+^. The effective diffusion coefficients were calculated as D_f_ = 1.72 × 10^−8^ and D_p_ = 1.75 × 10^−13^, although comparable data from previous studies are scarce. MB adsorption onto MT-ZLSH-Li^+^ aligns with previous research, which suggests that characteristics such as high surface area, pore volume, structural defects in the zeolite, and adsorbate-adsorbent interactions influence equilibrium times. This phenomenon is driven by the orientation of MB molecules at different adsorption sites before final adsorption is achieved [69]. MT-ZLSH-Li^+^ contains LTA, sodalite-hydroxysodalite, and cancrinite zeolitic phases, with an average pore size of 0.4 nm. However, during ion exchange processes, the pore size of zeolites can vary depending on the incorporated ion [70]. In the case of MT-ZLSH-Li^+^, the pore size remained unchanged. Given that the MB molecule has approximate dimensions of 1.7 nm × 0.76 nm × 0.33 nm, larger than the pore size of MT-ZLSH-Li^+^, the larger molecular size of MB can restrict the cation exchange of MB molecules within the cages and channels of zeolite [71]. MB adsorption likely depends on the availability and orientation of adsorption sites within the material structure, as MB adsorbs in three different molecular orientations [72]. The results suggest that intraparticle diffusion is one of the limiting mechanisms in MB adsorption onto MT-ZLSH-Li^+^, as the large molecular size of MB delays easy entry into the pores of MT-ZLSH-Li^+^. MB adsorption onto zeolites (NaA [73] and Na-Y [74]) has also shown than when large amounts of MB accumulated on the external surface, they blocked the entrances to the inner pores, thereby inhibiting further adsorption processes. Additionally, it is important to note that MT-ZLSH-Li^+^ was synthesized from a non-pure raw material, which may have introduced obstructions in the pores due to the chemical composition of mining tailings, further reducing the availability of sites for MB diffusion. Consistent with thermodynamic results, MB adsorption increases with temperature, indicating that MB diffusion into the pores is more favourable at higher temperatures. Given the pore size limitations of LTA zeolites, MB adsorption first occurred on the surface, followed by diffusion into the internal pores of MT-ZLSH-Li^+^, with restrictions as previously determined in similar systems. Thus, since the pore size of the adsorbent is a critical factor for larger molecules such as MB, obtaining larger pore sizes in zeolites to improve adsorption efficiency is highly desirable. Incorporating some experimental techniques during synthesis may help, but most of them are expensive and not environmentally friendly [75].

**Figure 7:**
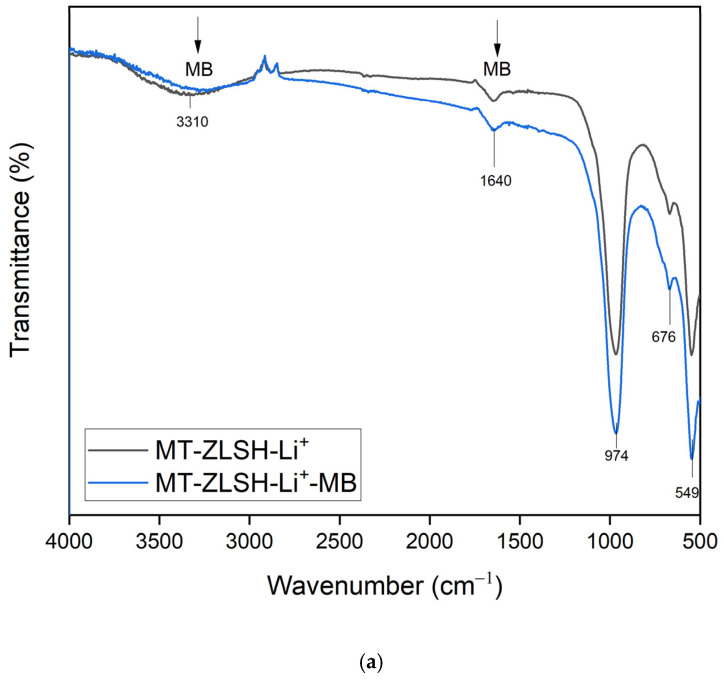

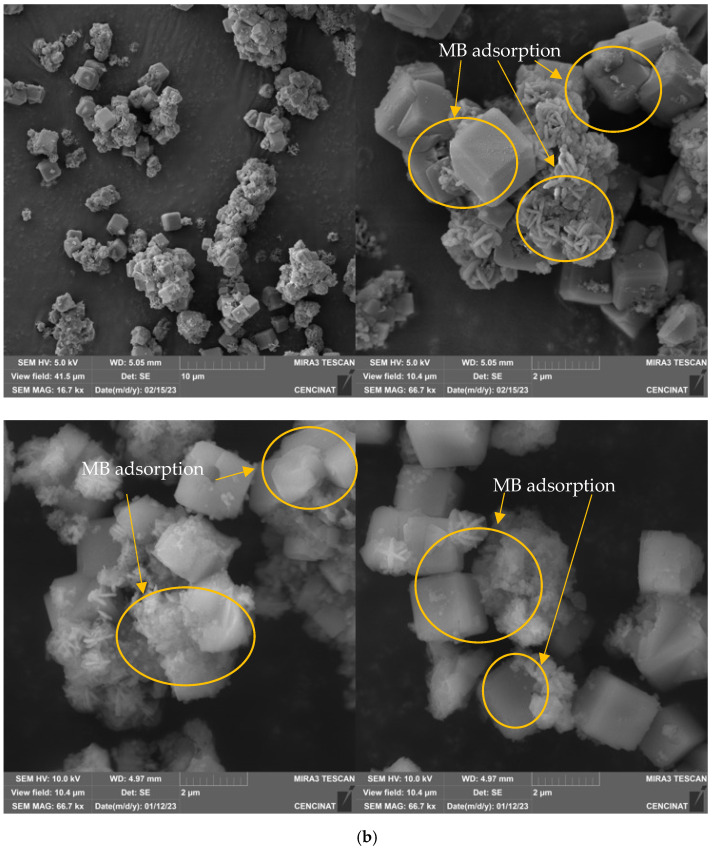
FTIR spectrum (**a**) and SEM micrographs (scale bar from 2 to 10 μm) (**b**) adsorbent after MB adsorption (MT-ZLSH-Li^+^-MB).

In the preliminary assessment of methylene blue (MB) adsorption capacity, MT-ZLSH-Li^+^ demonstrated a capacity of 42.6 mg·g^−1^, surpassing the parent zeolite MT-ZLSH, which exhibited a capacity of 29.7 mg·g^−1^. This increase in MB adsorption capacity on MT-ZLSH-Li^+^ correlated with a slight increase in surface area from 47.9 m^2^·g^−1^ to 51.5 m^2^·g^−1^. The synergistic effect of hydroxyl groups (Al-OH and Si-OH) from the zeolites (LTA, sodalite-hydroxysodalite, and cancrinite), combined with lithium exchange, significantly enhanced methylene blue adsorption compared to the unmodified synthesised zeolite. The potential of MT-ZLSH-Li^+^ for MB adsorption is further emphasized by its adsorption capacity, which was found to be 24 times greater than that for rhodamine B, which is another cationic dye. It also outperformed anionic dyes such as tartrazine and methyl orange by an impressive 41-fold [25]. The adsorption of methylene blue onto MT-ZLSH-Li^+^ can be explained by interactions revealed through the FTIR analysis (Figure 7a). Methylene blue contains functional groups such as OH (3450 cm^−1^), C=C (1385 cm^−1^), C=N (1590–1493 cm^−1^), and C–H (1170 cm^−1^), all of which actively participate during the adsorption process on MT-ZLSH-Li^+^. Notably, the primary absorption band of MT-ZLSH-Li^+^ at 3310, 1640, 974, 676, and 549 cm^−1^ exhibited noticeable shifts in both position and intensity. In particular, the changes observed at 1640 and 3310 cm^−1^ suggest the involvement of –OH functional groups in MB adsorption [64]. The literature strongly supports that MB adsorption onto zeolites is favoured by the presence of –OH groups, which participate in hydrogen bond formation. Specifically, the Si–O–H group plays a critical role in coordinating the lone pair of electrons of nitrogen atoms within the methylene blue structure, facilitating interaction. Additionally, ionic exchange, driven by the negative charges on the surface due to silanol and aluminol groups, has been identified as a key factor in MB adsorption [50]. In this study, the adsorption of MB was further enhanced by the introduction of lithium into MT-ZLSH-Li^+^ via ion exchange and the small amount of LiOH that dissociated into Li^+^ and OH^−^ species, forming additional active sites that are essential for ion exchange reactions, ultimately promoting higher MB adsorption capacity.

Following MB adsorption (Figure 7b), the morphology of MT-ZLSH-Li^+^ transformed into a thin film of small particles that enveloped the LTA cubes, sodalite lephispheres, hydroxysodalite spheres, and the lithium hydroxide and lithium carbonate layers, resulting in a rougher surface compared to the original MT-ZLSH-Li^+^ structure. However, it is also expected that some lithium species may have been lost during MB adsorption due to their high solubility in water. The changes observed in the morphology of MT-ZLSH-Li^+^ are attributed to the adsorption of methylene blue, which is consistent with previous studies on MB adsorption onto zeolites [58].

### 2.6. Desorption of Methylene Blue

The regeneration potential of MT-ZLSH-Li^+^ was evaluated, as it plays a crucial role in enabling reuse through multiple cycles of adsorption and desorption. Detailed information about the experimental adsorption–desorption process is provided in Table 5, with the outcomes dependent on the treatment medium (whether acidic or basic) used for the adsorbent. Notably, the desorption rate of MB significantly improved when MT-ZLSH-Li^+^ was treated in an acidic medium (HCl at pH 3), resulting in a recovery rate of 37%. This enhanced desorption is attributed to HCl weaking the hydrogen bonding interactions between the N–H functional group of MB and the surface OH^−^ groups of MT-ZLSH-Li^+^. Consequently, the increased electrostatic repulsion facilitated the easy release of methylene blue [50]. This finding aligns with previous studies, where the highest MB recovery from crosslinked zeolite was achieved under acidic conditions [76]. Unfortunately, limited information is available regarding the regeneration of zeolite adsorbents used for methylene blue removal.

The efficiency of MB adsorption onto MT-ZLSH-Li^+^ decreased during the initial cycle of adsorption–desorption. Notably, the treatment of MT-ZLSH-Li^+^ with HCl proved beneficial, as it increased porosity and surface area by removing inert mass, as documented in previous studies [77]. However, these findings suggest that MT-ZLSH-Li^+^ may not be a suitable adsorbent for continuous cycles of MB adsorption–desorption. The observed efficiency loss can be attributed to the potential blockage or loss of certain bonding sites after the desorption of MB from MT-ZLSH-Li^+^. In particular, lithium species and their functional groups can be lost during the adsorption process due to their high solubility in water.

The removal of MB using lithium-exchanged zeolite, derived from mining tailings as MT- ZLSH-Li^+^, has been compared to other adsorbents used for the same purpose, as summarized in Table S1 (Supplementary Materials). The adsorbent used in this study exhibits comparable, and in some cases even higher, removal efficiency than other adsorbents derived from zeolites and waste materials. However, other materials with superior characteristics have been reported to exhibit higher adsorption capacities than MT-ZLSH-Li^+^. Despite its limitations, MT-ZLSH-Li^+^ contains a moderate amount of active phase, which could be improved by exploring alternative synthesis method to increase surface area and thereby enhance its MB adsorption capacity. Currently, MT-ZLSH-Li^+^ stands as a promising and cost-effective material derived from ore mining tailings, offering a practical solution for water pollution control. MT-ZLSH-Li^+^ presents itself as a sustainable alternative, with potential contribution to environmental sustainability through industrial waste management and pollution control. This underscores the value of the developed material, serving as an important first phase in research.

### 2.7. Kinetics of Photocatalysis

**Figure 8:**
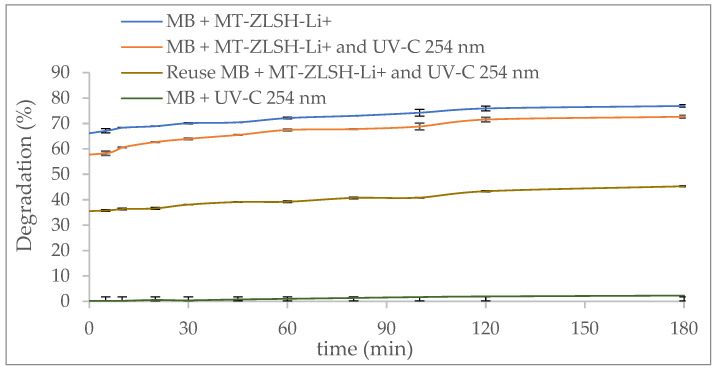
Photocatalytic activity of MT-ZLSH-Li^+^ in the degradation of methylene blue.

The photodegradation activity of MT-ZLSH-Li^+^ towards methylene blue (MB) is shown in Figure 8. The influence of UV light alone on methylene blue removal was around 2%, which can be attributed to the limited activity of organic dye under light irradiation [78]. This is comparable to previous studies, which reported 8% removal under UV-C light after 10 h [79]. In this study, the degradation of MB by MT-ZLSH-Li^+^ under UV-C light (254 nm) reached 77% within 180 min. The photocatalytic degradation rate was comparable to that of other zeolitic material used for methylene blue removal [67]. However, no comparable information was available for MB removal using lithium exchanged zeolite. The modest performance of MT-ZLSH-Li^+^ can be attributed to the limited concentration of lithium and other metallic species supported on the surface of the synthesized zeolite. The zeolite structure facilitated the adsorption of MB molecules, allowing greater accessibility of MB to the active species supported on the zeolite. This observation aligns with the physiochemical characterization discussed earlier, where the specific surface area of MT-ZLSH-Li^+^ increased by 8% compared to the parent zeolite MT-ZLSH. This increase in surface area significantly influences catalytic activity, as larger surface areas expose more active sites for both adsorption and catalytic reactions [80]. As shown in Figure 8, MB degradation reached 72.7% after 180 min due to adsorption alone, while photocatalytic degradation increased this to 76.9% (additional ~5% increase). This suggests that adsorption plays a crucial role in MB degradation, facilitating access to the limited number of metallic species on the zeolite surface, which enables the photocatalytic reaction. The high solubility of lithium species likely accounts for the low lithium incorporation on the zeolite (just 4.41 mg·g^−1^), consistent with previous reports on faujasite Y treated with LiOH. Despite the low lithium doping, the activity and selectivity of this alkaline-active component and its derivatives enabled reactions primarily on the external surface, limited by the small internal pores of the zeolite [81].

The experimental data for the photocatalytic degradation of methylene blue by MT-ZLSH-Li^+^ fit best with the pseudo-first-order kinetic model Equation (7), and the first-order rate constant (K_obs_, min^−1^) was evaluated [67].

(7) $$\ln\left(c_{a}/c_{t}\right)=K_{obs}t$$

where c_t_ (mg·L^−1^) represents the methylene blue concentration at time t after irradiation, and c_a_ (mg·L^−1^) is the MB concentration after the adsorption equilibrium. The calculated K_obs_ constant for MT-ZLSH-Li^+^ (Table 6) was lower than the previously reported first-order rate constant for other zeolitic materials used as catalysts [67]. This can be explained by the interactions of light with the surface of nanocatalysts, where electrons are excited from the valance band to the conduction band, producing radicals. The efficiency of photodegradation depends on the concentration of the dopant as well as factors such as photocatalyst size, bandgap energy, and surface area [82].

The literature describes the photocatalytic reactions caused by UV light exposure in the presence of zeolites containing metal species (M: Al, K, Ca, Fe, Zn, Ba, Li), which enable the degradation of methylene blue. For MT-ZLSH-Li^+^**,** this degradation follows the mechanisms outlined in Equations (8)–(15) for MT-ZLSH-Li^+^ [67,83]:

(8) $$\mathrm{Zeolite}-M+\mathrm{hv}\to \mathrm{Zeolite}-{M(e}_{\mathrm{CB}}^{-}{+h}_{\mathrm{VB}}^{+})$$

(9) $$e_{\mathrm{CB}}^{-}+O_{2}\to {O_{2}}^{-\cdot }$$

(10) $$H_{2}O+{O_{2}}^{-\cdot }\to OOH^{\cdot }+OH^{-}$$

(11) $$2OOH^{\cdot }\to O_{2}+H_{2}O_{2}$$

(12) $$H_{2}O{+h}_{\mathrm{VB}}^{+}\to OH^{\cdot }+H^{+}$$

(13) $$OH^{-}{+h}_{\mathrm{VB}}^{+}\to OH^{\cdot }$$

(14) $$MB{+h}_{\mathrm{VB}}^{+}\to MB^{+}$$

(15) $$MB^{+}+({O_{2}}^{-\cdot },OOH^{\cdot },OH^{\cdot })\to \mathrm{Products}(H_{2}O+CO_{2})$$

Superoxide $({O_{2}}^{-\cdot }$) and hydroxyl ($OH^{-}$) radicals play a pivotal role as reactive oxygen species (ROS) in the photooxidation of dye pollutants like MB. When the energy of incident light exceeds the catalyst’s band gap, the generation of electrons and holes promotes the breakdown of the dye through these reactive oxygen species [79]. As reported in previous studies, when UV light is absorbed, the excited methylene blue dye molecules can convert $O_{2}$ into ${O_{2}}^{-\cdot }$. This newly formed ${O_{2}}^{-\cdot }$ superoxide can then react with protons from water’s autoprotolysis, resulting in the formation of superoxide radicals $OOH^{\cdot }$ and hydroxyl ions $OH^{-}$ [67]. The effectiveness of MB degradation in these reactions is closely tied to the surface area of the catalyst [79]. These radicals are capable of fully degrading MB into carbon dioxide, water, and mineral acids. Zeolites are recognized as effective substrates for incorporating reactive species, enhancing photocatalytic activity by increasing surface area and preventing agglomeration. Furthermore, the polar nature of zeolites enables charge separation, allowing them to act as both electron $e^{-}$ acceptors and donors [84]. Typically, the photocatalytic degradation of methylene blue in the presence of zeolites and UV irradiation involves the separation of electron-hole pairs on the zeolite surface, leading to reduction and oxidation reactions. Electrons can be captured by adsorbed molecular oxygen species, while holes may be trapped by water or adsorbed methylene blue molecules. Consequently, methylene blue is decomposed into non-toxic products, such as water (H_2_O) and carbon dioxide (CO_2_), through photo-generated oxidants [67].

The role of the different lithium species (Li^+^, LiOH, and Li_2_CO_3_) present in MT-ZLSH-Li^+^ is now discussed. Lithium ions (Li^+^) incorporated within the zeolitic frameworks, alongside other metal ions, contribute to the charge balance of the zeolite, stabilizing the structure and influencing its electronic properties. This creates a more conducive environment that enhances charges separation and transfer, facilitating the mobility of photo-generated electrons and holes [83] and activating photocatalytic reactions. While lithium ions do not directly participate in photocatalysis, Li+ and other metal elements promote the adsorption of MB on the zeolite surface through electrostatic attraction [84]. Although not directly reactive, they improve pollutant accessibility to catalytic sites, indirectly supporting the photocatalytic process.

On the other hand, lithium species, such as LiOH and Li_2_CO_3_, located on the surface of MT-ZLSH-Li^+^ play a direct role in the degradation of MB in aqueous solutions. These species are exposed to both pollutants and light irradiation, driving the photocatalytic mechanism. LiOH dissociates into Li+ and OH-, increasing the availability of reactive oxygen species (ROS), particularly hydroxyl radicals (OH•) [83], which are responsible for the oxidative degradation of MB, as described in Equations (8)–(15), under light irradiation. Conversely, Li_2_CO_3_ has low solubility in water, remaining stable on the ZLSH-Li^+^ surface and participating indirectly in the photocatalytic process. Since the external surface of the zeolite is more active in photocatalysis than the internal framework, the presence of a thick Li_2_CO_3_ film may partially block light penetration, reducing access to the encapsulated metal species within the zeolite structure and limiting their involvement in photocatalytic reactions [85].

The band gap energy (E_g_) of MT-ZLSH-Li^+^ was calculated using Equation (16) [84]:

(16) $$E_{g}=\frac{1240}{\lambda_{max}}$$

where $\lambda_{max}$ represents the absorption wavelength (nm). Tauc’s equation Equation (17) was applied to determine E_g_ of the samples [67]:

(17) $${\left(\alpha h\nu \right)}^{n}=A\left(h\nu -E_{g}\right)$$

where α refers to the absorption coefficient, hν is the light frequency, n is 2 for direct or ½ for indirect transitions, and A is a constant. Direct allowed transitions are predominant in zeolite materials, so n = 2 was used.

**Figure 9:**
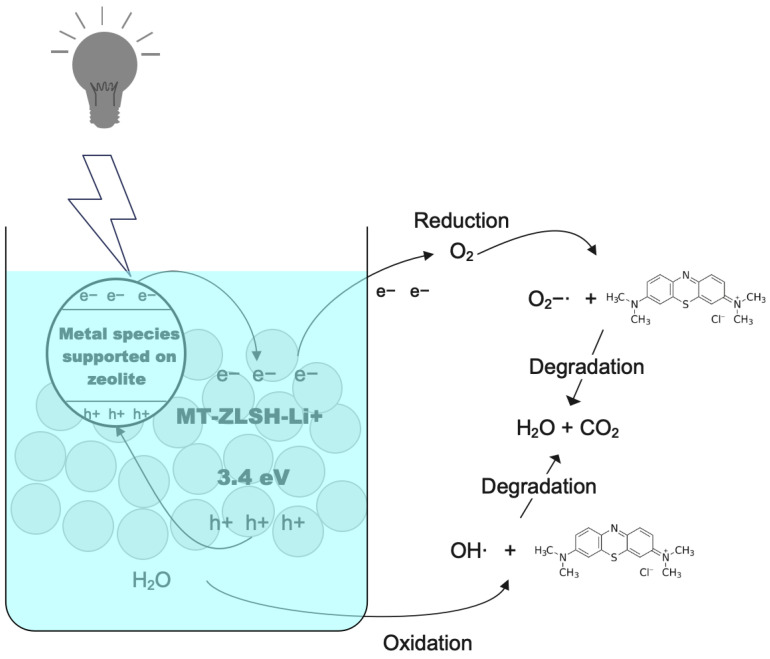
Photocatalytic mechanisms proposed for MB degradation on MT-ZLSH-Li^+^ catalyst.

The electrons transported from the metallic species of the zeolite to its framework extended the charge carrier lifetime. MT-ZLSH-Li^+^, when exposed to visible light, supports the formation of e^−^ — h^+^ pairs in the conduction band (CB) and valence band (VB). These photogenerated pairs (e^−^ — h^+^) move to the surface of MT-ZLSH-Li^+^, where they react with adsorbed species to form active radicals. $E_{VB}$ and $E_{CB}$ were calculated using Equations (18) and (19), respectively [84].

(18) $$E_{VB}=\chi -E^{C}+0.5E_{g}$$

(19) $$E_{CB}=E_{VB}-E_{g}$$

where χ represents the absolute electronegativity of both lithium-exchanged and single zeolite. $E^{C}$ is the free electron energy on the H-scale (NHE, 4.5 eV), and $E_{g}$ is the band gap of MT-ZLSH-Li^+^ and MT-ZLSH, determined to be 3.4 eV and 4.7 eV, respectively. The band gap of MT-ZLSH-Li^+^ is higher than that of graphitic carbon nitride (CN) treated with an LiOH catalyst ($E_{g}$ = 2.63 eV) [26], likely due to the lower photocatalytic activity of the zeolite substrate compared to graphitic carbon nitride materials. The calculated E_VB_ and E_CB_ potentials for MT-ZLSH-Li^+^ were −1.87 eV ($E_{VB}$) and −5.27 eV ($E_{CB}$), respectively. In comparison, MT-ZLSH exhibited $E_{VB}$ and $E_{CB}$ values of +4.18 eV and −0.52 eV, respectively. The CB level of MT-ZLSH-Li^+^ (−5.27 eV) is more negative than that of MT-ZLSH (−0.52 eV). According to previous reports, photoexcited electrons in the CB of metallic species are transferred to the CB of the zeolite via the inner electric field, facilitating reduction or oxidation reactions [84]. Meanwhile, the VB of MT-ZLSH (+4.18 eV) is more positive than that MT-ZLSH-Li^+^ (−1.87 eV), allowing h^+^ to transfer from the zeolite to the metal species, improving the separation of e^−^ — h^+^ pairs. This combined effect of the zeolite and supported metal species promotes efficient e^−^ — h^+^ transfer. MT-ZLSH zeolite exhibited poor electrical conductivity, but it remains an excellent electron acceptor-donor and transporter. Electrons from metallic species (e^−^ CB) could be transported across the zeolite surface, where they combine H_2_O and O_2_ molecules to produce hydroxyl radicals (OH) and superoxide anions (O_2_) [84]. Consequently, the adsorption of MB molecules onto the surface of MT-ZLSH-Li^+^ enhances MB degradation by facilitating electron transfer from the conduction (CB) to the valence band (VB) [83]. Surface-bound MB molecules are optimally positioned within the available adsorption sites on MT-ZLSH-Li^+^, resulting in heightened photoactivity (Figure 9). In contrast, when the dye remains suspended in solution, its interaction with reactive oxygen species is limited. A higher concentration of catalyst particles can enhance proton adsorption, promoting electron-hole generation and accelerating the formation of reactive oxygen species [79].

In the second photocatalytic cycle using MT-ZLSH-Li^+^, the removal efficiency of MB decreased, reaching only 45%. This decline can be attributed to the reduced density of photocatalytic activity centres due to the MB adsorbed on the MT-ZLSH-Li^+^ surface and the accumulation of degradation products from MB. Nevertheless, the material’s limited reusability highlights the need for improving the synthesis method to achieve better distribution and stability of the active phase.

The photodegradation performance of MB using lithium-exchanged zeolite (MT-ZLSH-Li^+^), derived from mining tailings, has been compared to other catalysts used for similar purposes, as summarized in Table 7. The photodegradation achieved with MT-ZLSH-Li^+^ is comparable to, and in some cases exceeds, other catalyst used for the same purpose. However, the time required to achieve this removal rate is significantly longer compared to alternative catalysts. This limited efficiency can be attributed to the relatively small surface area of MT-ZLSH-Li^+^, which restricts the amount of active phase supported on the zeolite.

### 2.8. Future Directions: Incorporating Lithium Waste into Adsorbent Materials

The incorporation of lithium into MT-ZLSH-Li^+^ significantly enhances its adsorption and photocatalytic activity. While pure lithium chemicals were used in this phase of the research, we acknowledge the high cost and strategic importance of lithium as a resource. In future studies, we aim to explore the use of lithium-containing waste from spent lithium-ion batteries as an alternative source. This approach would not only lower material costs but also address the issue of lithium waste management, promoting recycling and supporting a circular economy. The shift towards using waste-derived lithium would further enhance the sustainability of MT-ZLSH-Li^+^ as an adsorbent material for industrial applications. Additionally, a comprehensive cost-benefit analysis to assess the feasibility of using lithium from waste streams must be performed in future investigations.

## 3. Materials and Methods

### 3.1. Reagents

The reagents used in this study included NaOH (EMSURE^®^. Darmstadt, Germany), NaAlO_2_ (Sigma-Aldrich. St. Louis, MO, USA), and methylene blue hydrate (Sigma-Aldrich. USA). The synthesis of zeolite from mining tailings and the incorporation of lithium were carried out under conditions described in our previous report [25]. The zeolite (MT-ZLSH) was synthesized with the following molar ratios: SiO_2_/Al_2_O_3_ = 12; Na_2_O/SiO_2_ = 2 and H_2_O/Na_2_O = 100. Specific amounts of NaOH and NaAlO_2_ were dissolved in deionized water and mixed with 5 g of mining tailing. The solution was stirred for 30 min at room temperature. The mixture was treated by alkaline fusion for 4 h at 800 °C. The resulting solid was mixed with deionized water for 1 h and then aged for 30 h. The mixture was subsequently treated hydrothermally for 17 h at 90 °C to crystallize the zeolite. The synthesized zeolite was washed until the pH decreased between 7 and 8 and then dried at 90 °C for further testing. For lithium incorporation, 30 g of MT-ZLSH zeolite was refluxed at 40 °C in 250 mL of a LiCl (0.1 M) solution, adjusted to 12 by adding NaOH. The solutions turned slightly whitish, and after 6 h of refluxing with constant stirring, the suspension was evaporated, leaving a solid that was washed repeatedly with deionized water using a microfiltration membrane (average pore size 0.1 μm) until no chloride compounds were detected. The resulting MT-ZLSH-Li^+^ was dried and calcined at 200 °C for 4 h.

### 3.2. Materials Characterization

The X-ray fluorescence (XRF) analysis of the MT-ZLSH and MT-ZLSH-Li^+^ samples was performed in a Bruker Titan 800 handheld XRF analyser (Bruker, Billerica, MA, USA). The lithium content was determined by an atomic Absorption Spectrometer AAA (Perkin Elmer, Waltham, MA, USA) and inductively coupled plasma optical emission spectroscopy (ICP-OES, Optima 8000 Perkin Elmer, Waltham, MA, USA). The contents of Na, Fe, Ca, K, and Zn were determined with the AAA, while Li, Si, and Al were analysed with ICP-OES in the lithium solution at initial and equilibrium states. Morphology was analysed using a scanning electron microscopy (SEM) Tescan Mira 3 field emission scanning electron microscope (Brno, Czech Republic). The X-ray diffraction (XRD) analysis was performed using a D8 Advance A25 diffractometer (Bruker, Karlsruhe, Germany), with a Cu Kα anode radiation source (λ = 0.1542 nm) operating at 40 kV and 40 mA. Diffraction patterns were recorded between 4° and 50° at 2θ. Infrared absorption spectra were recorded between 4000 and 500 cm^−1^ using a FTIR transform spectrometer at 50–60 Hz (Nicolet iS10, 4100 Jasco, Easton, MD, USA). KBr was used to prepare a tablet sample, and the spectra were obtained by collecting 32 scans using a 4 cm^−1^ resolution. Nitrogen adsorption was used to determine the specific surface area of the adsorbents with an automatic adsorption analyser (Micrometrics Chemisorb 2720, Norcross, GA, USA) using nitrogen gas adsorption by the single-point method. The pH drift method was applied to determine the point of zero charge (pH_PZC_), equilibrating 0.1g of MT-ZLSH-Li^+^ in deionized water and 0.01 N and 0.05 N NaCl solutions ranging from pH 2 to pH 11 for 24 h, after which the final pH was recorded [86]. The optical properties of the prepared materials were evaluated by means of UV–vis DRS within the range of 240 to 900 nm based on the conventional method reported elsewhere [67,84].

### 3.3. Methylene Blue Adsorption Assays

Standard methylene blue (MB) solutions (1000 mg·L^−1^) were prepared in deionized water. Adsorption assays were conducted by equilibrating 25 mL of a 20 mg·L^−1^ MB solution at pH 7 with 0.1 g of MT-ZLSH or MT-ZLSH -Li^+^ for 24 h. The supernatant was separated via centrifugation at 5000 rpm and further filtration through 0.45 μm. The pH and concentration of methylene blue were determined in the liquid phase. Methylene blue concentrations were measured using a UV-vis spectrometer at a wavelength of 664 nm. The concentration of the MB dye was calculated from a constructed calibration curve using UV-Vis spectrophotometer data ranging from 0.5 to 5 mg L^−1^. The methylene blue adsorption capacity at equilibrium was determined using Equation (20).

(20) $$q_{e}=\frac{{v\times (c}_{0}{-c}_{e})}{w}$$

$q_{e}$ is the equilibrium adsorption capacity (mg·g^−1^), v is the solution volume (L), c_0_ and c_e_ are the initial and equilibrium MB concentrations (mg·L^−1^), and w is the adsorbent mass (g).

#### 3.3.1. Adsorption as Function of the pH

The effect of pH on MB adsorption was studied using solutions with pH values ranging from 3 to 11. Methylene blue solutions (25 mL and 5 mg·L^−1^) were equilibrated with 0.1 g of MT-ZLSH-Li^+^ for 24 h. The pH and concentration of MB were determined in the liquid phase, and adsorption capacity was determined according to Equation (20).

#### 3.3.2. Kinetic of Methylene Adsorption

Adsorption kinetics were investigated by equilibrating 0.1 g of MT-ZLSH-Li^+^ with 25 mL of a 20 mg·L^−1^ MB solution at pH 7. Samples (5 mL) were withdrawn at specific time intervals (t) ranging from 15 s to 24 h. The pH and concentration of methylene blue were determined in the liquid phase, and adsorption capacity at specific times was calculated using Equation (21).

(21) $$q_{t}=\frac{{v\;\times \;(c}_{0}{-c}_{t})}{w}$$

$q_{t}$ is the adsorption capacity at time t (mg·g^−1^), and $c_{t}$ is the concentration at time t (mg·L^−1^ MB). Experimental data were fitted to the pseudo-first order Equation (22), pseudo-second order kinetic model Equation (23), and intraparticle diffusion model Equation (24), which is a kinetic model based on diffusion in the methylene blue solution and further diffusion in the sphere-shaped adsorbent.

(22) $$\ln\left(q_{e}-q_{t}\right)=\ln\left(q_{e}\right)-k_{1}t$$

(23) $$\frac{t}{q_{t}}=\frac{1}{k_{2}q_{e}^{2}}+\frac{t}{q_{e}}$$

where $k_{1}$ (h^−1^) and $k_{2}$ (g·mg^−1^·h^−1^) are kinetics constants.

(24) qt=ktt12+A

where $k_{t}$ (mg·g^−1^·h^−1/2^) is the intraparticle diffusion rate constant, and A (mg·g^−1^) is a constant that provides information about the thickness of the boundary layer.

If liquid film diffusion ($D_{f}$) governs the adsorption rate in the film phase, the process is well described by Equation (25). If particle diffusion ($D_{p}$) controls the process, the adsorption rate is described by Equation (26) [87].

(25) $$-\ln\left(1-\left(\frac{q_{t}}{q_{e}}\right)\right)=\frac{D_{f}c_{s}}{hrc_{z}}t$$

(26) $$-\ln\left(1-{\left(\frac{q_{t}}{q_{e}}\right)}^{2}\right)=\frac{2\pi^{2}D_{p}}{r^{2}}t$$

where $c_{s}$ (mg·L^−1^) and $c_{z}$ (mg·kg^−1^) are MB concentrations in solution and in adsorbent, respectively, r is the average radius of the adsorbent particle (particles below 200 mesh ≈ radius: 3.7 × 10^−5^ m), t is the contact time (min), and h is the film thickness of the adsorbent particle (1 × 10^−5^ m for a poorly stirred solution).

#### 3.3.3. Isotherms of Methylene Blue Adsorption

MT-ZLSH-Li^+^ was equilibrated in 25 mL of methylene blue solution at concentrations ranging from 1 to 300 mg·L^−1^ at pH 7. The experimental data were fitted to both the Freundlich and Langmuir isotherm models. The Freundlich isotherm model describes a heterogeneous surface with diverse adsorption sites according to the linear expression in Equation (27) [88].

(27) $$\log q_{e}=\log k_{F}+\frac{1}{n}\log c_{e}$$

where: q_e_ is the adsorbate content in the solid phase at equilibrium (mg·g^−1^), K_F_ is the affinity constant (L·mg^−1^), and c_e_ is the concentration of adsorbate in the liquid phase at equilibrium (mg·L^−1^), with n being the Freundlich constant. The Langmuir isotherm model assumes monolayer adsorption of the adsorbate onto a homogenous adsorbent surface according to the linearized expression in Equation (28) [89].

(28) $$\frac{c_{e}}{q_{e}}=\frac{c_{e}}{q_{m}}+\frac{1}{k_{L}q_{m}}$$

where: q_m_ is the maximum adsorption capacity (mg·g^−1^), and k_L_ is the affinity constant (L·mg^−1^). $r_{L}$ is the favourability of the adsorption process according to Equation (29):

(29) $$r_{L}=\frac{1}{{1+K}_{L}c_{o}}$$

#### 3.3.4. Thermodynamics of Methylene Blue Adsorption

The experimental data were used to calculate thermodynamic parameters such as Gibbs free energy ΔG^0^ (kJ·mol^−1^), enthalpy ΔH^0^ (kJ·mol^−1^), and entropy ΔS^0^ (kJ·mol^−1^) using van’t Hoff Equation (30) [90].

(30) $$\ln k_{c}=\frac{-\Delta H^{0}}{R}\times \frac{1}{T}+\frac{\Delta S^{0}}{R}$$

where $k_{L}$ (L·mg^−1^) is the Langmuir constant. The dimensionless parameter $k_{c}$ is obtained by multiplying $k_{L}$ by the molecular weight of the adsorbate (M_w_, g·mol^−1^) and applying the factor of 1000, representing the number of moles of pure water per litre, as described in Equation (31) [64]. R is the universal gas constant (8.314 J mol^−1^·K^−1^), and T is the absolute temperature (K).

(31) $$k_{c}=k_{L}\times M_{w}\times 1000\times 1$$

#### 3.3.5. Desorption of Methylene Blue

A methylene blue solution (25 mL, 20 mg·L^−1^) at pH 7 was equilibrated for 24 h with 0.1 g MT-ZLSH-Li^+^. The supernatant was separated by centrifugation at 5000 rpm and further filtered through a 0.45 μm membrane. The solid phase was washed multiple times with deionized water and stored for further testing. The pH and concentration of methylene blue were determined in the liquid phase. Solutions of NaOH at pH 11 and HCl at pH 3 were prepared and equilibrated for 24 h with the recovered solid phase. The concentration of methylene blue was measured in the liquid phase, and a new adsorption cycle was carried out.

### 3.4. Photocatalytic Degradation of Methylene Blue

The photocatalytic activity of MT-ZLSH-Li^+^ was evaluated for degradation of methylene blue, as lithium-based materials have shown effectiveness in degrading organic compounds due to their oxidative capacity when exposed to light [91]. A methylene blue solution (100 mL, 15 mg·L^−1^) at pH 7 was tested by photodegradation using 0.05 g of MT-ZLSH-Li^+^. The suspension was stirred in the dark for 30 min to allow an adsorption–desorption equilibrium between MB and the photocatalyst. Afterward, the suspension was irradiated with UV light while stirring continuously. A 5 mL sample was withdrawn at 30 min in the dark, and subsequent samples of the same volume were collected at intervals between 5 and 360 min while the suspension was irradiated with a simulated solar lamp at wavelengths between 300 and 800 nm. The pH of the solution was continuously measured, and the concentration of MB was measured in the liquid samples. The effect of photolysis on methylene blue degradation was also verified.

## 4. Conclusions

In this study, we explored the incorporation of lithium onto the surface of a zeolite synthesized from mining tailings. Comprehensive physicochemical characterizations were performed on both MT-ZLSH and MT-ZLSH-Li^+^. Methylene blue (MB) removal from synthetic wastewater was achieved through adsorption and photocatalytic processes. The MT-ZLSH zeolite exhibited three distinct zeolitic phases (LTA, sodalite-hydroxysodalite, and cancrinite) with remarkable crystallinity. The introduction of lithium led to changes in functional groups and surface properties as well as the formation of nanometric hydroxysodalite and cancrinite phases, enhancing MB adsorption efficiency. Notably, efficient MB adsorption was achieved within the pH range typical of real wastewater, indicating the practical applicability of this material as an adsorbent. MB adsorption onto MT-ZLSH-Li^+^ was governed by both physical and chemical adsorption mechanisms, including ion exchange, electrostatic attraction, and hydrogen boding Thermodynamic studies confirmed the exothermic nature of MB adsorption onto MT-ZLSH-Li^+^. The pseudo-second order and intraparticle diffusion models best described the MB adsorption data, revealing distinct adsorption stages. The kinetics of the process were effectively explained by initial film diffusion followed by intraparticle diffusion, with the latter being the limiting mechanism in MB adsorption. The photocatalytic test on MT-ZLSH-Li^+^ demonstrated 77% degradation of MB within 180 min. However, a decline in performance was observed during the second cycle of both adsorption and photocatalysis, likely due to the reduction in photocatalytic active centres caused by MB adsorption and the accumulation of degradation products on the MT-ZLSH-Li^+^ surface. In comparison with the other catalyst, MT-ZLSH-Li^+^ exhibited competitive performance in MB photodegradation, though the time required to reach similar degradation rates was longer. This limited efficiency can be attributed to the small surface area and the restricted amount of active phase on the zeolite. While MT-ZLSH-Li^+^ shows potential for efficient dye removal from industrial wastewater via adsorption, further improvements are needed to develop a more effective adsorbent. The aim of this study was to produce an active phase for photocatalytic purposes with a higher content, better distribution, and increased surface area as well as improved long-term stability. Nevertheless, the material obtained may be more suitable for use in non-aqueous media due to the high solubility of lithium compounds, which presents a significant limitation for pollutant removal from water.

## Figures and Tables

**Table 1:** Chemical composition of the synthesized MT-ZLSH and modified zeolite MTA-ZLSH-Li^+^.

	Major Elements *								
ID	Al_2_O_3_ (%)	SiO_2_ (%)	P_2_O_5_ (%)	S (%)	K_2_O (%)	CaO (%)	Fe_2_O_3_ (%)	ZnO (%)	BaO (%)
MT-ZLSH	18.1	26.1	ND	0.3	0.1	1.7	2.5	0.2	<lq ^1^
MT-ZLSH-Li^+^	21.2	28.4	<lq	0.2	0.0	1.9	2.7	0.2	1.3
	Trace Elements *								
ID		Co_3_O_4_ ppm	CuO ppm	ZnO ppm	As_2_O_3_ ppm	Ba ppm	PbO ppm	MnO ppm	
MT-ZLSH		0.06	0.05	0.06	0.02	0.07	0.02	0.11	
MT-ZLSH-Li^+^		0.06	0.05	0.06	0.02	0.07	0.02	0.11	

* Elements were determined via X-ray fluorescence (XRF) analysis. ^1^: below the limit of quantification.

**Table 2:** Isotherm parameters for MB adsorption onto MT-ZLSH-Li^+^.

Temperature K	Langmuir			Freundlich		
	$q_{m}$ (mg·g^−1^)	$k_{L}$ (L·mg^−1^)	R^2^	1/n	$k_{F}$ (mg·g^−1^)	R^2^
293.7	23.4	0.24	1.00	0.50	3.07	0.78
299.1	24.8	0.24	1.00	0.50	3.45	0.73
304.2	28.8	0.20	0.99	0.49	3.97	0.72

**Table 3:** Thermodynamic parameters for MB adsorption onto MT-ZLSH-Li^+^.

Temperature (K)	R^2^	ΔG^0^ (kJ·mol^−1^)	ΔS^0^ (kJ·mol^−1^·K^−1^)	ΔH^0^ (kJ·mol^−1^)
293.7	0.93	−27.4	0.06	−10.6
299.1		−28.0		
304.2		−28.0		

**Table 4:** Kinetic parameters of MB adsorption on MT-ZLSH-Li^+^.

Model	Kinetic Parameters	MT-ZLSH-Li^+^
Pseudo-first order	q_e_ (mg·g^−1^)	22.61
	k_1_ (h^−1^)	0.18
	R^2^	0.85
Pseudo-second order	q_e_ (mg·g^−1^)	37.26
	k_2_ (g·mg^−1^·h^−1^)	0.001
	R^2^	0.98
Intraparticle diffusion	k_t1_ (mg·g^−1^.s^−1/2^)	361.05
	R^2^	0.95
	k_t2_ (mg·g^−1^·s^−1/2^)	114.13
	R^2^	0.72
	k_t3_ (mg·g^−1^·s^−1/2^)	1.64
	R^2^	0.95
	k_t4_ (mg·g^−1^·s^−1/2^)	2.93
	R^2^	0.94
HPDF film diffusion	D_f_ (m^2^·s^−1^)	1.72 × 10^−8^
	R^2^	0.85
HPDM particle diffusion	D_p_ (m^2^·s^−1^)	1.75 × 10^−13^
	R^2^	0.89

**Table 5:** Methylene blue adsorption and desorption efficiency.

#	Solution	pH	$q_{e}$ 1st _cycle_ (mg·g^−1^)	$q_{des}$ (mg·g^−1^)	$q_{e}$ 2nd _cycle_ (mg·g^−1^)
1	NaOH	11	29.3 ± 0.1	1.1 ± 5.7	6.1 ± 1
2	HCl	3	29.1 ± 0.3	10.7 ± 0.0	25.5 ± 1

**Table 6:** Kinetic parameters of the photocatalytic degradation of methylene blue over MT-ZLSH-Li^+^.

Zeolite	R^2^	K_obs_ (min^−1^)
MT-ZLSH-Li^+^	0.97	0.003

**Table 7:** Comparative analysis of methylene blue photodegradation among various catalysts.

Catalyst	Degradation	K_obs_ (min^−1^)	Reference
Lithium exchanged zeolite obtained from mining tailing MT-ZLSH-Li^+^	77%/180 min	0.003	This study
Zeolite synthesized using aluminium isopropoxide	24.85%/150 min	0.012	[67]
Zeolite synthesized using sodium aluminate	83.28%/60 min	0.013	
Zeolite supported CdS/TiO_2_/CeO_2_ composite	99.9%/120 min	~0.0201	[84]
Reduced graphene oxide (rGO)–metal oxide (TiO_2_/Fe_3_O_4_) based nanocomposites	100%/5 min	0.56	[7]
Chabazite exchanged Ag AgCHA	71.37%/120 min	0.0066	[83]
Chabazite exchanged Cu CuCHA	98.92%/120 min	0.0266	

## Data Availability

Data are contained within the article.

## Supplementary Materials

The following supporting information can be downloaded at: https://www.mdpi.com/article/10.3390/molecules29194643/s1; Table S1. Comparative analysis of methylene blue adsorption efficiency among various adsorbents.
