# Supplementary material for: A Novel Approach to Waste Recycling and Dye Removal: Lithium-Functionalized Nanoparticle Zeolites
Source: Molecules. 2024 Sep 29;29(19):4643. doi: 10.3390/molecules29194643 (PMC11478182; doi:10.3390/molecules29194643)
Supplement: Supplementary file 1 [file molecules-29-04643-s001.zip › molecules-3232266-supplementary.pdf]

Article

A Novel Approach to Waste Recycling and Dye Removal: Lithium-Functionalized Nanoparticle Zeolites

Diana Guaya [1,*], Alexis Debut [2] and Jhuliana Campoverde [1]

1 Departmento de Química, Universidad Técnica Particular de Loja, Loja 110107, Ecuador; jncampoverde2@utpl.edu.ec
2 Centro de Nanociencia Nanotechnología, Universidad de las Fuerzas Armadas ESPE, Sangolquí 171103, Ecuador; apdebut@espe.edu.ec
* Correspondence: deguaya@utpl.edu.ec

Table S1. Comparative analysis of methylene blue adsorption efficiency among various adsorbents.

Adsorbents	Langmuir q_m ($mg \cdot g^{-1}$)	Pseudo – second order q_e ($mg \cdot g^{-1}$)	Reference
Lithium exchanged zeolite obtained from mining tailing MT-ZLSH-Li^+	23.4	37.23	This study
Alginate beads impregnated with magnetic Chitosan@Zeolitenanocomposite	6.14	1.08	[77]
Coal fly produced analcime zeolite	16.53	7.15	[9]
Coal fly produced cancrinite zeolite	27.05	5.99	
Magnetic graphene oxide modified zeolite composites Cu-Z-GO-M (1:1)	94.48	93.78	[10]
CZ (ZSM-5 zeolite commercially)	75.53	29.91	[64]
NZ (hydrothermally nanosheet MFI-synthesized zeolite)	227.70	91.74	
Zwitterion composite chitosan-epichlorohydrin/zeolite	165.1	123.7	[58]
Chitosan-activated clinoptilolite biocomposite	143.67	85.90	[48]
Red mud waste into mesoporous ZSM-5	270.27	48.54	[51]
